# Temporal dynamics of frontoparietal processing during personal space intrusion

**DOI:** 10.1016/j.isci.2026.116829

**Published:** 2026-07-16

**Authors:** Sijia Xiang, Xinbo Zou, Chuqing Luo, Ning Liu

**Affiliations:** 1 State Key Laboratory of Cognitive Science and Mental Health, Institute of Biophysics, Chinese Academy of Sciences, Beijing 100101, China; 2 College of Life Sciences, University of Chinese Academy of Sciences, Beijing 100049, China; 3 School of Life Sciences, University of Science and Technology of China, Hefei, China

**Keywords:** personal space, EEG, alpha rhythms, low-gamma rhythms, granger causality

## Abstract

Understanding the neural basis of personal space (PS) is crucial for elucidating the mechanisms of social behavior. We investigated EEG responses in healthy adults viewing approaching faces designed to simulate intrusions into PS. Approaching faces elicited stronger right N170 modulation than static faces, with weaker effects for non-face objects. Time-frequency analyses revealed early alpha-power decreases over the frontal region of interest (ROI), followed by later alpha-power decreases and gamma-power increases over the parietal ROI, selectively for approaching faces. Motion information was decoded earlier in the parietal ROI than in the frontal ROI, with the frontal ROI showing face-specific sensitivity. We further demonstrated sustained parietal-to-frontal low-gamma feedforward signaling during face approach, with late frontal-to-parietal alpha-band feedback. Crucially, feedforward signaling predicted individual PS size. Together, these findings suggest that coordinated frontoparietal interactions may facilitate rapid detection and continuous monitoring of PS intrusions, providing a mechanistic framework for adaptive social defense.

## Highlights

• Approaching faces elicit stronger right N170 modulation
• Alpha suppression and low-gamma enhancement mark PS-relevant face approach
• Sensor-level GC reveals bidirectional parietal-frontal interactions
• Parietal-to-frontal low-gamma signaling relates to individual PS size

## Graphical abstract

**Figure:**
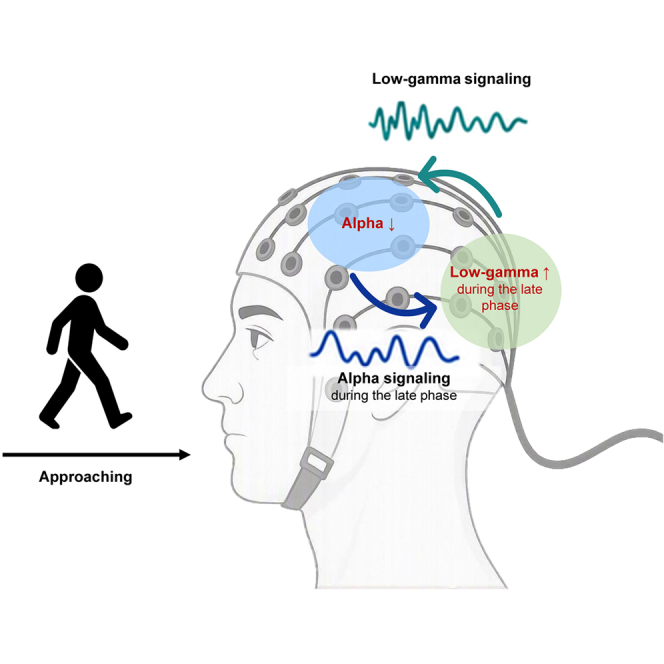

## Teaser

Neuroscience; Cognitive neuroscience

## Introduction

Personal space (PS) refers to the area surrounding an individual, into which intrusion by others typically causes discomfort.^1^ Humans regulate PS automatically during social interactions. The size of PS is shaped by multiple factors. Observer-related characteristics, such as gender, age, personality traits, cultural background, and social status, can influence PS size.^2^^,^^3^^,^^4^ Approacher-related features, including familiarity and facial expression, also modulate PS.^5^^,^^6^^,^^7^^,^^8^ Situational context further plays a critical role. For instance, individuals tend to enlarge their PS when confronted with perceived threats.^8^^,^^9^ Thus, PS serves not only as a protective boundary against potential threats^10^ but also as a facilitator of social connection and intimacy.^11^ Abnormalities in PS regulation have been observed in various mental disorders characterized by social dysfunction, such as schizophrenia,^12^^,^^13^ borderline personality disorder,^14^ autism spectrum disorder,^15^^,^^16^^,^^17^ and social anxiety disorder.^9^ Understanding the neural bases of PS is therefore critical for explaining fundamental mechanisms of social behavior.

Functional magnetic resonance imaging (fMRI) studies in humans have identified PS-related activity within the frontoparietal network, particularly in the dorsal intraparietal sulcus (DIPS) and ventral premotor cortex (PMv).^12^^,^^13^^,^^14^^,^^18^^,^^19^ These regions respond preferentially to approaching faces versus withdrawing faces, with no such bias observed for non-face objects, highlighting their roles in PS processing.^18^ Furthermore, resting-state fMRI has revealed that functional connectivity (FC) between DIPS and PMv correlates with individual PS size.^18^ In patients with schizophrenia, the DIPS exhibits an exaggerated response to approaching faces, with activity correlating with both PS size and symptom severity.^12^

Despite these advances, the temporal dynamics of neural activity during PS intrusion, as compared with intrusion by non-social stimuli, have yet to be fully elucidated. In particular, it remains unclear how neural activity evolves over time across different frequency bands and how information is dynamically exchanged within the frontoparietal network during intrusion. Electroencephalography (EEG), with its high temporal resolution, provides a powerful and non-invasive tool to address this gap. However, few EEG studies have targeted PS directly, with most primarily focusing on the modulatory effects of facial expressions on neural responses to PS. For example, previous studies have shown that the N170 component, a negative potential peaking around 170 ms after stimulus onset, is significantly more negative in response to approaching upright fearful faces compared to approaching neutral faces and inverted stimuli.^20^ Similarly, the P1 component, a positive peak occurring at 90 to 120 ms over posterior occipital sites, is more positive when viewing approaching angry faces.^21^^,^^22^ At the oscillatory level, alpha-band reduction is greater when viewing a person in near versus far space, and stronger for an approaching versus receding experimenter, suggesting sensitivity to interpersonal proximity.^23^ Yet, because non-social objects were not included as controls in these studies, the specificity of these findings for PS remains unclear. More importantly, the potentially distinct roles of key PS regions (i.e., PMv and DIPS) and the dynamics of the information flow within the frontoparietal network during PS intrusion have not been established. Answering these questions can yield important insights into how PS is represented and regulated in the brain, with potential clinical relevance.

In the present study, we employed EEG to characterize the temporal dynamics of neural activity underlying PS processing. Consistent with previous studies,^18^^,^^24^ approaching face stimuli were used to simulate PS intrusion. We compared neural responses to approaching versus static stimuli across faces and non-face categories to investigate whether and how the frontoparietal network preferentially supports PS processing. We then used effective connectivity analysis to characterize the direction of information flow during face approach and also examined correlations between neural activity and subjective discomfort distance (DD).

## Results

### Behavioral results

**Figure 1:**
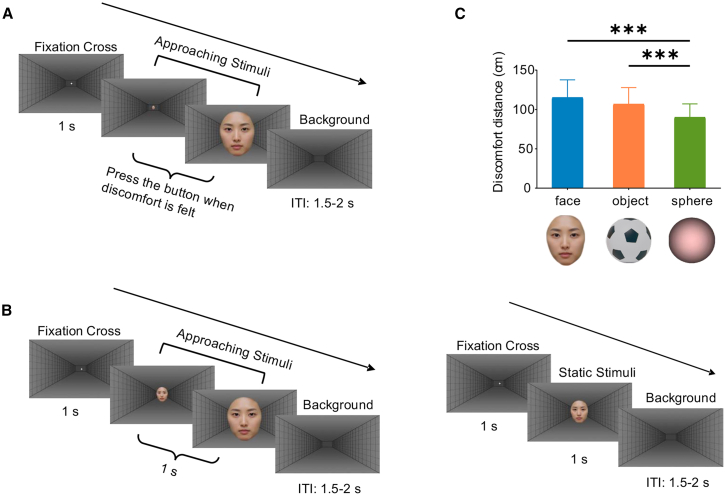
Experimental paradigms and behavioral results (A) Behavioral paradigm. Participants viewed approaching stimuli and pressed the spacebar when they felt uncomfortable. (B) EEG paradigm. Participants passively viewed videos of either approaching or static stimuli. Participants did not respond during the main trials but were required to press the spacebar when red squares appeared during occasional catch trials, thereby ensuring sustained attention. (C) Behavioral results showing mean discomfort distances for each stimulus category (faces, objects, spheres). Error bars represent 95% confidence intervals (CIs). Statistical significance was assessed using Friedman tests followed by Dunn’s post hoc tests (*n* = 28 participants). ∗∗∗*p* < 0.001.

We measured the DD for the three stimulus categories (face, object, sphere) and found that no participant exceeded a conventional threshold (|Z| > 2.5; maximum Z = 2.50), indicating the absence of extreme statistical outliers by standard criteria. Then, we conducted a Friedman test to examine the main effect of Category on DD (Figure 1C). The results revealed a significant difference among categories (χ^2^(2) = 31.84, *p* < 0.001). Post hoc comparisons using Dunn’s test showed that DDs were significantly greater for faces (M = 115.13 cm, SD = 11.00 cm) and objects (M = 106.77 cm, SD = 10.27 cm) than for spheres (M = 90.04 cm, SD = 8.30 cm) (faces: Cohen’s d = 2.09, *p* < 0.001; objects: Cohen’s d = 1.27, *p* < 0.001). No significant difference was found between faces and objects (Cohen’s d = 0.38, *p* = 0.482). These findings indicate that participants maintained larger DD for real-world stimuli (faces and objects) compared to virtual spheres, with no significant differences observed between faces and objects.

### ERP results

We conducted ERP analyses focusing on three components: P1, N170, and the vertex positive potential (VPP). For each component, we performed two-way repeated-measures ANOVAs on component amplitudes with Motion (approaching, static) and Category (face, object, sphere) as within-subject factors (see Table 1).

**Table 1:** ANOVA results for ERP components with motion and category as within-subject factors

		P1		N170		VPP
		L	R	L	R	
Main effect of motion	df1	1	1	1	1	1
	df2	27	27	27	27	27
	F	0.142	1.042	10.879	14.444	0.277
	*p*	0.709	0.316	0.003∗∗	0.001∗∗	0.603
	partial *η*^*2*^	0.005	0.037	0.287	0.349	0.01
Main effect of category	df1	1.528	1.538	1.605	1.542	2
	df2	41.253	41.517	43.339	41.624	54
	F	2.751	6.152	28.419	40.410	41.030
	*p*	0.089	0.008∗∗	<0.001∗∗∗	<0.001∗∗∗	<0.001∗∗∗
	partial *η*^*2*^	0.092	0.186	0.513	0.599	0.603
Interaction	df1	1.973	1.960	1.676	1.668	2
	df2	53.274	52.923	45.256	45.042	54
	F	0.029	0.811	0.892	3.426	0.195
	*p*	0.971	0.448	0.401	0.049∗	0.823
	partial *η*^*2*^	0.001	0.029	0.032	0.113	0.007

∗*p* < 0.05, ∗∗*p* < 0.01, and ∗∗∗*p* < 0.001.

For the P1 component (Figures S1A–S1C), which reflects early visual processing,^25^ neither the main effect of Motion nor the interaction between Motion and Category reached significance for amplitudes at either the left or right occipital ROI (Figures S1D and S1E). However, there was a significant main effect of Category on the right P1 amplitude, with objects eliciting larger responses than faces (Cohen’s d = 0.72, *p* = 0.002) (Figure S1E).

**Figure 2:**
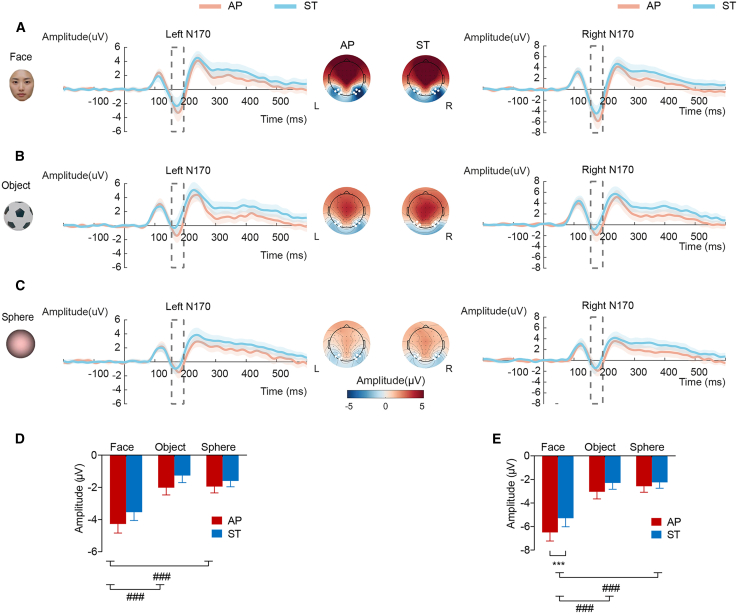
Event-related potential (ERP) results for the N170 (A–C) Grand average ERP waveforms for the left and right N170 in response to faces (A), objects (B), and spheres (C). Shaded areas represent 95% CIs. Topographical maps show scalp distributions averaged over the 175–215 ms time window (dashed box in waveforms), with the locations of the electrodes analyzed indicated by white dots. (D and E) Mean amplitudes of the left (D) and right (E) N170 for each condition. Error bars indicate the standard error of the mean (SEM). # indicates follow-up post hoc tests for the main effect of Category; ∗ denotes follow-up post hoc tests for the interaction effect between Category and Motion, comparing approaching and static conditions. ###*p* < 0.001 and ∗∗∗*p* < 0.001; *n* = 28 participants, Bonferroni-corrected for multiple comparisons. AP: approaching; ST: static; L: left, R: right.

Analysis of the N170 amplitudes yielded consistent findings across both hemispheres (Figures 2A–2C). Significant main effects were observed for both Motion and Category (Table 1). Post hoc comparisons showed that approaching stimuli evoked larger N170 amplitudes than static stimuli (left: Cohen’s d = 0.62, *p* = 0.003; right: Cohen’s d = 0.72, *p* = 0.001). Note that the pattern in the left hemisphere was qualitatively similar but attenuated relative to the right hemisphere, which is consistent with prior reports of right-lateralized N170 responses.^5^ Moreover, faces elicited greater amplitudes than both objects (left: Cohen’s d = 1.42, *p* < 0.001; right: Cohen’s d = 1.35, *p* < 0.001) and spheres (left: Cohen’s d = 0.98, *p* < 0.001; right: Cohen’s d = 1.28, *p* < 0.001), with no significant difference between the latter two (left: Cohen’s d = 0.09, *p* = 1.000; right: Cohen’s d = 0.16, *p* = 1.000). Notably, a significant interaction between Motion and Category was found for the right N170 amplitude (Figure 2E), but substantially smaller for the left one (Figure 2D). Further analyses revealed that, in the right hemisphere, approaching faces elicited significantly larger amplitudes compared to static faces (Cohen’s d = 0.81, *p* < 0.001), whereas the effects for objects and spheres were weaker (objects: Cohen’s d = 0.46, *p* = 0.064; spheres: Cohen’s d = 0.25, *p* = 0.590). These findings suggest that the right N170 component may be more sensitive to the approach of faces as compared to non-face stimuli.

For the VPP component (Figures S2A–S2C), we found a significant main effect of Category on amplitude (Table 1). Post hoc tests showed that faces elicited stronger responses than objects (Cohen’s d = 1.21, *p* < 0.001), which in turn elicited stronger responses than spheres (Cohen’s d = 0.69, *p* = 0.003) (Figure S2D). Neither the main effect of Motion nor the interaction between Motion and Category was significant for VPP amplitude.

### Decoding results

**Figure 3:**
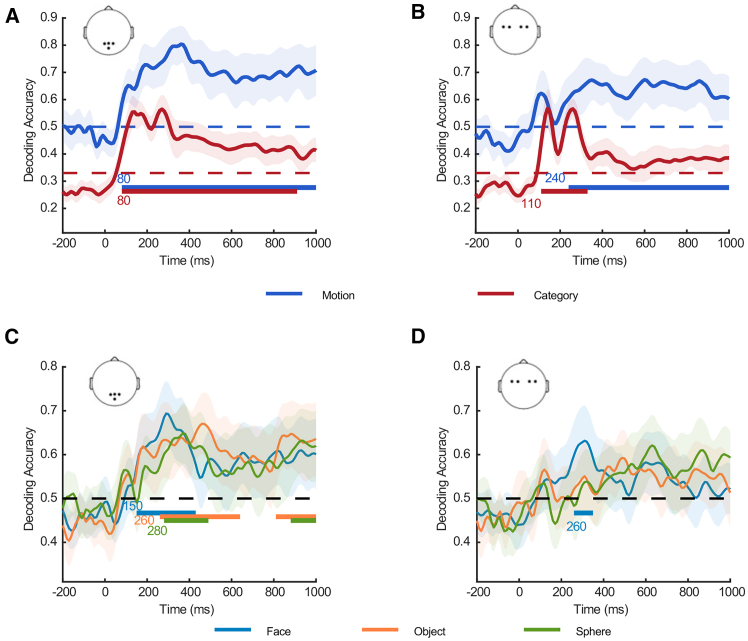
Decoding accuracy of motion and category representation in parietal and frontal regions of interest (ROIs) (A and B) Decoding accuracy over time for motion (approaching vs. static) and category (face, object, sphere) in the parietal (A) and frontal (B) ROIs. (C and D) Decoding accuracy over time for motion (approaching vs. static) separated by stimulus category in the parietal (C) and frontal (D) ROIs. Numbers adjacent to the colored horizontal bars indicate the onset time (in milliseconds) of statistically significant decoding above chance level. The dashed line indicates chance-level performance. Shaded areas represent 95% CIs. Colored horizontal bars mark periods of significant above-chance decoding, determined by cluster-based permutation tests (*n* = 28 participants; *p* < 0.01, one-tailed).

To examine the dynamic neural representations of motion and category information, we conducted decoding analyses. As shown in Figure 3A, the parietal ROI exhibited significantly above-chance decoding accuracy for both motion and category information beginning at 80 ms post-stimulus (*p* < 0.01, one-tailed). In contrast, the frontal ROI showed above-chance decoding for category information from 110 ms (*p* < 0.01, one-tailed) and for motion information from 240 ms (*p* < 0.01, one-tailed) (Figure 3B). These findings suggest that the parietal ROI can rapidly process both motion and category information, while in the frontal ROI, category information may be detected earlier than motion, with both emerging later than in the parietal ROI.

To further examine differences in processing approaching versus static stimuli within each category, we conducted additional decoding analyses within the two ROIs. In the parietal ROI, above-chance decoding accuracy was observed for all three categories, with the earliest significant time point for faces (150 ms, *p* < 0.01, one-tailed), followed by objects (260 ms, *p* < 0.01, one-tailed) and spheres (280 ms, *p* < 0.01, one-tailed) (Figure 3C). In contrast, the frontal ROI showed above-chance decoding between approaching and static conditions only for faces, emerging later than in the parietal ROI and reaching significance at 260 ms (*p* < 0.01, one-tailed; Figure 3D). These findings highlight the specificity and rapidity of neural processing for approaching faces compared to non-face objects.

### Time-frequency results

**Figure 4:**
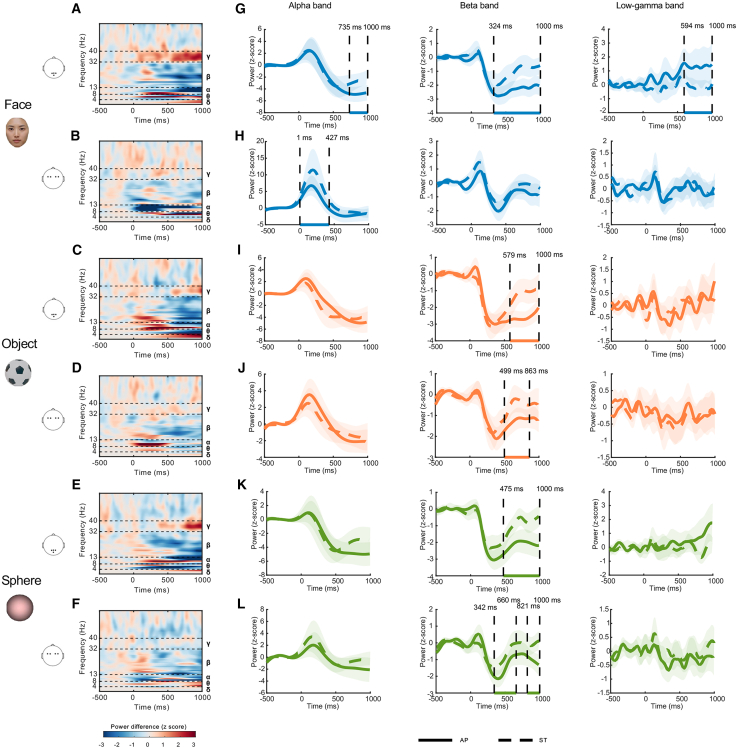
Time-frequency analyses of alpha, beta, and low-gamma power in parietal and frontal ROIs (A–F) Time-frequency spectrograms contrasting approaching vs. static stimuli in parietal and frontal ROIs for faces (A and B), objects (C and D), and spheres (E and F). (G–L) Alpha (left column), beta (middle column), and low-gamma (right column) power averaged over parietal and frontal ROIs as a function of time for faces (G and H), objects (I and J), and spheres (K and L). Solid lines represent the approaching condition; dashed lines represent the static condition. Shaded areas represent 95% CIs. Colored lines overlaid on the x axis highlight time points with significant differences between approaching and static conditions (*n* = 28 participants; two-sided paired t-tests, cluster-based permutation correction, *p* < 0.05, 50,000 permutations).

To characterize how neural oscillations differentiate between approaching and static stimuli, time-frequency analyses employing Morlet wavelet transforms were conducted for each stimulus category within the parietal and frontal ROIs. As shown in Figures 4A–4F, differences among the three categories were observed across a range of frequencies. Differences in lower frequencies largely corresponded to the conventional alpha and beta bands, whereas in the higher-frequency range, effects were confined to a statistically significant cluster spanning 32–40 Hz. No comparable effects were observed in higher gamma ranges (Figure S3). Accordingly, subsequent analyses focused on the alpha and beta bands, together with this data-informed low-gamma range. Results for the remaining bands are shown in Figure S4.

In the alpha band, approaching faces elicited a significant decrease in spectral power compared to static faces in the frontal ROI during an early time window (1–427 ms, *p* = 0.024; Figure 4H left), and a similar, nearly significant decrease was observed in the parietal ROI during a later window (735–1000 ms, *p* = 0.054; Figure 4G left). No significant differences were found between approaching and static conditions for objects or spheres (Figures 4I–4L). These findings suggest that alpha-band changes may be particularly sensitive to approaching faces.

Across all three categories, approaching stimuli elicited a significant decrease in beta power compared to static stimuli in the parietal ROI during a later time window (faces: 324 to 1000 ms, *p* = 0.001, Figure 4G middle; objects: 579 to 1000 ms, *p* = 0.003; Figure 4I middle; spheres: 475 to 1000 ms, *p* = 0.005; Figure 4K middle). Notably, this effect was observed only for objects (499–863 ms, *p* = 0.016; Figure 4J middle) and spheres (342–660 ms, *p* = 0.023; 821 to 1000 ms, *p* = 0.044; Figure 4L middle) but not for faces (Figure 4H middle) in the frontal ROI.

In the low-gamma band, approaching faces evoked significantly increased spectral power relative to static faces in the parietal ROI during a later time window (594–1000 ms, *p* = 0.001; Figure 4G right). Again, no notable differences were observed for objects or spheres (Figures 4I–4L right).

Together, these results indicate that power changes in both the alpha and low-gamma bands may be especially responsive to approaching faces, whereas beta-band changes seem to be more sensitive to approaching non-face objects.

### GC results

**Figure 5:**
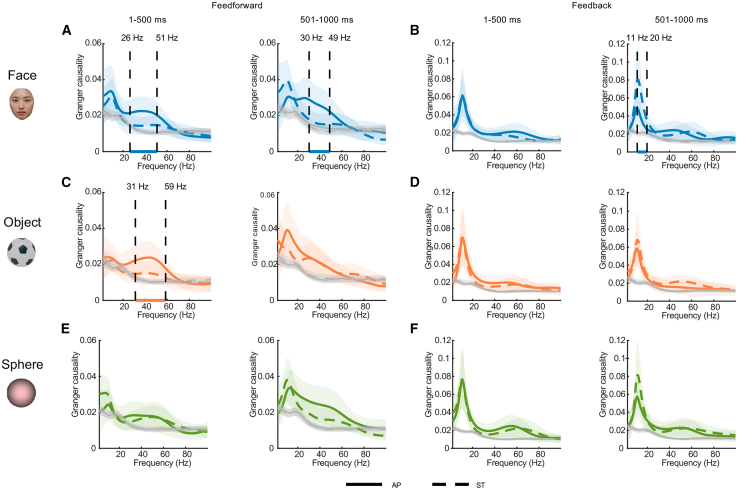
Granger causality (GC) analysis between parietal and frontal ROIs (A and B) Spectral GC for feedforward (A) and feedback (B) information in response to faces. (C and D) Spectral GC for feedforward (C) and feedback (D) information in response to objects. (E and F) Spectral GC for feedforward (E) and feedback (F) information in response to spheres. Solid (approaching) and dashed (static) lines show mean spectral GC; shading indicates 95% CIs. Colored lines overlaid on the x axis mark frequencies with significant differences between approaching and static conditions (*n* = 28 participants; two-sided paired t-tests, cluster-based permutation correction, *p* < 0.05, 50,000 permutations). Gray lines represent 99.9% CIs from permutation testing by randomly shuffling the time series (1,000 randomizations), and the corresponding shaded areas indicate 95% CIs.

Next, we examined the effective connectivity between the frontal and parietal ROIs associated with face approach in the PS context. GC analysis was employed to assess the directional interactions between the frontal and parietal ROIs. Spectral GC profiles, along with 99.9% confidence intervals derived from permutation testing (1,000 random shuffles of the time series), are presented in Figure 5. Based on the results of the time-frequency analyses, data were divided into two time windows: 1–500 ms (early window) and 501–1000 ms (late window).

In the feedforward direction (from the parietal ROI to the frontal ROI), the early window showed stronger GC for approaching compared to static conditions for both faces (26–51 Hz, *p* = 0.032; Figure 5A left) and objects (31–59 Hz, *p* = 0.011; Figure 5C left) but not spheres (Figure 5E left). In the late window, only approaching faces evoked significantly stronger GC than static faces (30–49 Hz, *p* = 0.047; Figure 5A right). In the feedback direction (from the frontal ROI to the parietal ROI), a narrow peak around 10 Hz was observed. Additionally, there was a nearly significant reduction in GC for approaching faces compared to static faces in the late window (11–20 Hz, *p* = 0.052; Figure 5B right). Notably, these bands largely overlapped with the frequency bands showing significant spectral power differences between faces and non-face objects. When analyses were restricted to these bands, we also found significant differences in GC between approaching and static conditions (low-gamma feedforward connectivity in the early window: faces, *p* = 0.007, objects, *p* = 0.015; low-gamma feedforward connectivity for faces in the later window: *p* = 0.016; alpha feedback connectivity for faces in the later window: *p* = 0.018). Our findings indicate that the dynamic connectivity between the parietal and frontal ROIs may be particularly sensitive to approaching faces.

To directly assess whether our findings could be driven by the well-known influence of volume conduction, which can introduce instantaneous mixing of signals across channels, we conducted a time-reversed GC analysis, a commonly used control for testing the validity of directional inferences.^26^^,^^27^^,^^28^ In this procedure, if the observed directionality were primarily driven by instantaneous mixing or noise structure, similar effects would be expected after reversing the temporal order of the signals.^26^ However, as shown in Figure S5, the originally observed directional effects were no longer present in the time-reversed data. This result suggests that our findings may not be trivially explained by zero-lag signal spread and instead reflect temporally structured dependencies.^28^

### Correlations between neural activity and behavior

**Figure 6:**
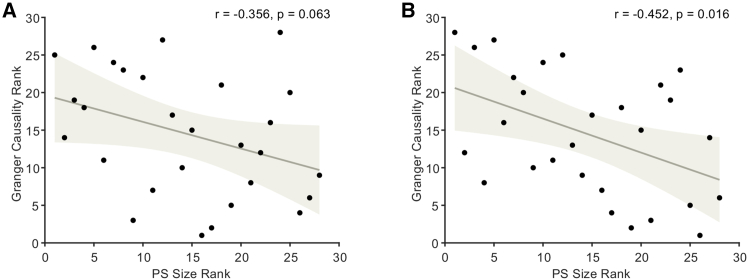
Correlations between GC and personal space (PS) size (A and B) Correlations between PS size and GC from the parietal to frontal ROI within the gamma-band during both the early window (26–51 Hz, A) and the late window (30–49 Hz, B). Dashed lines indicate 95% CIs. Correlations were assessed with two-tailed Spearman’s rank tests, with *n* = 28 participants.

To investigate the relationship between neural activity and behavior, we conducted correlational analyses between frontoparietal connectivity and PS size. Based on the above results, we specifically examined correlations between PS size and GC from the parietal ROI to the frontal ROI both in the early window (band: 26–51 Hz) and the late window (band: 31–59 Hz) (Figure 6), as well as GC from the frontal ROI to the parietal ROI in the late window (band: 11–20 Hz).

In the approaching face condition, GC from the parietal ROI to the frontal ROI showed a significant correlation with PS size, particularly during the late window (the early window: r = −0.356, *p* = 0.063, two-tailed, Figure 6A; the late window: r = −0.452, *p* = 0.016, two-tailed; Figure 6B). No similar correlations emerged for GC from the frontal ROI to the parietal ROI (r = 0.163, *p* = 0.409). Notably, no significant correlations were observed for objects or spheres. These findings suggest that stronger feedforward connectivity from the parietal ROI to the frontal ROI may be associated with a smaller PS, indicating greater tolerance for close interpersonal proximity.

## Discussion

In this study, we found that the right N170 component showed differential responses to approaching faces. Approaching faces also elicited distinct oscillatory changes: alpha-band power in the frontal ROI decreased during the early phase, whereas low-gamma power in the parietal ROI increased during the late phase. Temporal decoding analysis further revealed that motion information for faces was decoded earlier in the parietal ROI than in the frontal ROI. Furthermore, GC analyses demonstrated significantly enhanced low-gamma band feedforward influences and decreased alpha-band feedback influences during face approach. Additionally, the strength of the feedforward connectivity correlated with PS size. Note that these findings were specific to approaching faces and were not observed for non-face stimuli. Our results directly address gaps in previous PS studies, providing evidence on how the frontoparietal network dynamically and specifically processes social versus non-social approach signals.

### ERP and oscillatory responses to face approach

Our ERP analyses showed that the occipital P1 component was not significantly modulated by motion or by the interaction of motion and stimulus category, consistent with previous findings.^21^ Both the N170 and VPP components are well-established markers of face processing.^29^^,^^30^^,^^31^ A simple main effect of category would not, by itself, support a PS-specific interpretation. Therefore, our ERP analyses went beyond this canonical face effect by focusing on within-category contrasts, specifically comparing approaching versus static conditions for each stimulus type. This approach helps control for intrinsic differences in visual complexity and category sensitivity. Within this framework, approaching faces elicited a stronger modulation of the N170 than static faces, particularly in the right hemisphere, whereas this effect was markedly weaker for non-face objects. Thus, the key finding is not the presence of a face-sensitive N170 per se, but the additional modulation induced by approach (motion), which appears selectively amplified for socially relevant stimuli. This pattern may reflect early-stage sensitivity to socially relevant approach cues. The absence of modulation in the VPP suggests that it may be less sensitive than the N170 to dynamic aspects of face processing relevant to PS.

Previous studies have shown that N170 amplitudes are more negative when viewing approaching individuals with fearful facial expressions relative to neutral expressions, and for angry compared to neutral faces in virtual reality paradigms simulating approach.^20^^,^^32^ Thus, the N170 may play a key role in detecting potential social threats during face approach that simulates PS intrusion. Additionally, the stronger effects observed in the right hemisphere align with evidence of the right hemisphere’s dominance in spatial attention, particularly within near space.^33^ Taken together, our findings suggest that the right N170 may reflect the initial neural processes of threat evaluation within the PS context.

We also conducted time-frequency analyses and found distinct oscillatory dynamics that differentiate faces from non-face stimuli. In particular, alpha power decreased in the frontal ROI during the early stage and in the parietal ROI during the later stage of exposure to approaching faces relative to static faces. Alpha reduction has been widely recognized as a marker of increased attentional engagement.^34^^,^^35^^,^^36^^,^^37^ Our results align with prior studies indicating greater alpha reduction in the occipital sites when individuals imagine humans approaching compared to inanimate objects,^38^ and stronger alpha reduction in the parietal sites when participants observe a person positioned in near versus far space.^23^ Nonetheless, the specificity of these alpha oscillation changes to PS processing remains contentious, particularly in the absence of non-social controls or differentiation between key PS-related regions (i.e., the frontoparietal network). By including objects and spheres as control stimuli, our findings indicate that alpha-band reduction is specifically associated with social, PS-related processing. Furthermore, the temporal pattern of alpha reduction—emerging first at frontal sites and later at parietal sites—indicates that attention allocation during face approach may be modulated by top-down mechanisms, consistent with previous studies on visual attention showing that alpha oscillations exert top-down influences.^39^

Furthermore, we observed higher low-gamma power in the parietal ROI during the late phase of approaching faces compared to static ones. It has been demonstrated that low-gamma band oscillations are closely associated with higher cognitive functions.^40^^,^^41^ For example, previous studies have shown that faces and facial features evoke stronger gamma activity (25–45 Hz) than non-face objects (e.g., watches) across multiple brain regions.^42^ The increased low-gamma activity in the parietal ROI we observed likely reflects heightened cognitive processing related to detecting the spatial location of socially relevant stimuli—a recognized function of the parietal cortex.^43^^,^^44^ Notably, the late phase of the approach approximately corresponds to the period during which the stimulus is calibrated to traverse behaviorally estimated discomfort boundaries. Therefore, such increased low-gamma activity may represent heightened spatial monitoring in response to a PS intrusion.

### Dynamic frontoparietal interactions during face approach

Our time-domain decoding analyses revealed that the parietal ROI successfully decoded motion information across all three stimulus categories, with earlier decoding for faces than for the other categories. In contrast, the frontal ROI showed significant decoding accuracy only for motion information from faces, and at a later latency than the parietal ROI. These results indicate that both ROIs are particularly sensitive to approaching faces. Importantly, our findings suggest that the parietal cortex may serve as an early detector of PS intrusions and relay this information to the frontal cortex.

GC analyses provided additional insight into the directional flow of information between these ROIs. Consistent with the decoding results, we observed enhanced feedforward low-gamma band influence from the parietal ROI to the frontal ROI during face approach. These functional results align with established anatomical connections within the frontoparietal network,^45^^,^^46^ wherein the parietal cortex provides critical visuospatial information for motor planning and execution in the PMv.^10^ Thus, our findings support a bottom-up mechanism in which the parietal cortex may deliver spatial information about approaching faces to the frontal cortex. Notably, the late-stage stronger feedforward low-gamma band activity was specific to faces and absent for non-face objects, suggesting that bottom-up communication may become particularly pronounced after PS is intruded and may prime preparatory motor responses, such as defensive or stepping away actions mediated by the PMv. Our findings are also consistent with electrophysiological studies in nonhuman primates (NHPs), which show that the space around the body is monitored by an interconnected parietal-frontal network.^10^ In NHPs, the ventral intraparietal area (VIP) within the parietal cortex is primarily involved in processing sensory information about nearby space, whereas the precentral gyrus (PZ) in the frontal cortex is more directly engaged in generating defensive motor outputs. The PZ receives most of its sensory input from VIP and influences movement through projections to multiple motor structures. Consistent with this organization, findings in NHPs support a model in which information converges in the VIP to represent nearby space, is transmitted to the PZ, and is transformed into motor commands that produce appropriate defensive actions.^10^^,^^43^ While these studies primarily address peripersonal space rather than interpersonal PS, the convergence at the level of frontoparietal organization provides a useful comparative framework. In contrast, the human literature has predominantly relied on fMRI to localize the regions involved, with relatively limited characterization of the temporal dynamics and interactions underlying these processes. In this context, our findings provide temporally resolved evidence of how such frontoparietal processes unfold during approaching stimuli in humans. Notably, our results fit well within the model derived from NHPs, suggesting that similar frontoparietal mechanisms—linking spatial monitoring in the parietal cortex with motor preparation in the frontal cortex—may underlie PS intrusion processing in humans. We also identified a negative correlation between the feedforward connectivity and PS size. This finding parallels the results of Holt et al.,^12^^,^^18^ who reported that weaker FC between the DIPS and PMv was associated with larger PS. Together, these previous studies and our findings provide converging evidence that the observed EEG responses are meaningfully related to PS-relevant approach processing. Importantly, our results extend this work by specifying the directionality of this connectivity.

Note that, in addition to feedforward signaling, we detected a feedback influence from the frontal ROI to the parietal ROI at ∼10 Hz during the late stage of approaching faces. This observation is consistent both with our finding that alpha reduction to approaching faces emerges first in the frontal ROI and subsequently in the parietal ROI, and with the well-established role of alpha oscillations in top-down attentional modulation.^39^ Together, these results suggest the presence of frontal-to-parietal top-down control related to attention. Importantly, this feedback influence was more pronounced in the late stage— approximately corresponding to the period during which the stimulus is designed to enter PS—and, combined with our low-gamma oscillation findings (increased power in the parietal ROI during the late stage), may contribute to heightened spatial monitoring in the parietal areas in response to a PS violation. In sum, our findings extend previous evidence from NHPs by demonstrating that the human frontoparietal network may implement both feedforward and feedback mechanisms, enabling effective monitoring of PS intrusion and preparation for appropriate defensive or stepping away actions.

**Figure 7:**
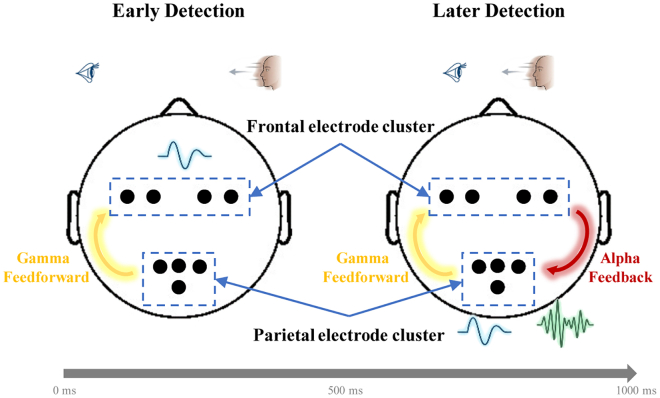
Schematic diagram of frontal-parietal dynamics during face approach Alpha-band power in the frontal electrode cluster decreases during the early phase, whereas low-gamma power in the parietal electrode cluster increases during the late phase. This pattern is accompanied by enhanced low-gamma feedforward influences from the parietal to the frontal electrode cluster during face approach, along with reduced alpha-band feedback influences during the late phase of the approach.

Taken together, our findings provide valuable insights into the distinct neural responses evoked by approaching faces compared with objects, supporting the existence of specialized neural mechanisms underlying PS. Importantly, our results also reveal the temporal dynamics of PS processing, underscoring the continuous interaction between parietal and frontal ROIs (Figure 7). Overall, these findings provide converging evidence for differentiated and time-resolved neural dynamics underlying PS, while offering a framework for future research into PS and its disturbances in mental disorders.

### Limitations of the study

Several limitations should be considered. First, although the sample size was determined *a priori* and is comparable to prior EEG studies, it may limit sensitivity to detect small-to-moderate effects. Future studies with larger samples will be important to more precisely estimate effect sizes and clarify the relative contributions of low-level visual dynamics and higher-level social processing. Second, no concurrent distance judgments or trial-by-trial subjective reports were collected during the main EEG trials. This design minimized response- and motor-related confounds, particularly over frontal electrodes, but did not provide a direct trial-by-trial index of subjective PS intrusion during EEG recording. Future studies could combine EEG with minimally motoric subjective or physiological measures to further characterize the subjective dynamics of PS intrusion during face approach. Third, the interpretation of connectivity results should be made with caution. While control analyses were conducted, sensor-level EEG measures remain susceptible to volume conduction, which may inflate apparent inter-regional dependencies. Therefore, future work using source-level analyses or multimodal approaches will be important to further validate these findings.

## Resource availability

### Lead contact

Requests for further information and resources should be directed to and will be fulfilled by the lead contact, Ning Liu (liuning@ibp.ac.cn).

### Materials availability

This study did not generate new unique reagents.

### Data and code availability

• Behavioral and EEG data reported in this paper are available from the lead contact upon reasonable request.
• Original analysis code used in this study is available from the lead contact upon reasonable request.
• Any additional information required to reanalyze the data reported in this paper is available from the lead contact upon reasonable request.

## Acknowledgments

The authors would like to thank Dr. Yang Fang for her help during data collection and analysis. This work was supported by STI2030-Major Projects (grant nos. 2021ZD0204200 and 2021ZD0200200) and the 10.13039/501100001809National Natural Science Foundation of China (grant no 32071094).

## Author contributions

S.X. and N.L. designed the research. S.X., X.Z., and C.L. performed the experiments. S.X. analyzed the data. S.X. and N.L. wrote the draft. S.X., X.Z., C.L., and N.L. reviewed and edited the paper.

## Declaration of interests

The authors declare no competing interests.

## STAR★Methods

### Key resources table

**Table:** 

REAGENT or RESOURCE	SOURCE	IDENTIFIER
**Software and algorithms**		

MATLAB R2020a	MathWorks	https://www.mathworks.com/
Adobe Photoshop	Adobe	https://www.adobe.com/
Presentation 23.1	Neurobehavioral Systems	https://www.neurobs.com/
EEGLAB 13.0	Swartz Center for Computational Neuroscience	https://sccn.ucsd.edu/eeglab/
GraphPad Prism 8	GraphPad Software	https://www.graphpad.com/
SHINE toolbox	Willenbockel et al.^47^	https://doi.org/10.3758/BRM.42.3.671
Neural Decoding Toolbox 1.0.4	Meyers^48^	http://www.readout.info
LIBSVM 3.25	Chang and Lin^49^	https://www.csie.ntu.edu.tw/∼cjlin/libsvm/
Multivariate Granger Causality toolbox	Barnett and Seth^50^	https://users.sussex.ac.uk/∼lionelb/MVGC/

### Experimental model and study participant details

#### Participants

Thirty-three right-handed adults (17 males, 16 females; age range: 19-28 years; mean (M) = 22.94, standard deviation (SD) = 2.73) were initially recruited for this study. All participants reported no history of neurological or psychiatric disorders, had normal or corrected-to-normal vision, and were free from color blindness or other ophthalmological conditions. To minimize confounding factors, participants confirmed that they had not consumed any medications, alcohol, tobacco, or caffeinated beverages prior to the experiment. Five participants were excluded due to poor signal quality (*n* = 1) or insufficient engagement (*n* = 4), resulting in a final sample of 28 individuals (14 males, 14 females; age range: 19-28 years; M = 22.89, SD = 2.77). All participants were unaware of the study’s aims prior to participation. Each provided written informed consent, completed a basic demographic questionnaire, and received monetary compensation upon completion of the study. This research was approved by the Institutional Review Board of the Institute of Biophysics, Chinese Academy of Sciences (2018-IRBH-001).

### Method details

#### Experimental stimuli

The visual stimuli comprised videos depicting both approaching and static presentations across three categories: faces, objects, and spheres. Face stimuli featured neutral expressions from four unfamiliar individuals (two males, two females), with images cropped to remove hair and ears and presented as elliptically contoured upper halves.^51^ In the present study, we conducted a pilot experiment and selected object stimuli to reduce large differences between faces and objects in perceived discomfort, thereby minimizing potential confounds between neural responses and substantial differences in subjective boundary. Object stimuli consisted of footballs and volleyballs, selected to match the faces in shape and size. The sphere stimuli were virtual objects created in Photoshop and included to control for low-level visual expansion in the absence of cues that support inference about an object’s real-world size, following prior studies.^18^ As noted in that study, a simple, uniformly textured sphere cannot be reliably assigned a position in depth because its absolute size cannot be inferred from prior knowledge or spatial context. As a consequence, such stimuli provide minimal support for estimating the approach trajectory or proximity.^52^ In contrast, faces and real-world objects (e.g., balls) are associated with well-established size expectations, which enable observers to interpret changes in image size as meaningful cues to approach in space. Four exemplars were included for both the object and sphere categories. All stimuli were standardized to ensure equal diagonal dimensions across categories. Four exemplars per category were used to increase variability and reduce habituation effects. To maintain visual consistency, all stimuli were luminance-matched using the SHINE toolbox^47^ and presented against a spatially graded gray background that was luminance-matched and included depth cues.^21^ For privacy protection, AI-generated faces created with the Mid-Journey model were used only for illustration (https://docs.midjourney.com/hc/en-us, see Figure 1A).

Stimuli were presented as videos at 25 frames per second. In the behavioral experiment, each video lasted 5 seconds, with approaching stimuli expanding in visual angle from 3.1 ° × 3.1 ° (corresponding to a distance of 5.5 m) to 17.2 ° × 17.2 ° (0.5 m). Based on the behavioral results of DD measurement, the EEG experiment utilized shorter videos lasting 1 second, in which approaching stimuli expanded from 5.8 ° × 5.8 ° (1.5 m) to 19.1 ° × 19.1 ° (0.5 m), covering the transition from outside to inside DD. In the present study, viewing distance was simulated through controlled changes in visual angle, based on the standard geometric relationship between an object’s physical size and its retinal projection. Specifically, given a fixed viewing distance between the participant and the display (60 cm), we used established visual angle calculations to determine the on-screen size corresponding to different implied distances. We note that the reported distances (in meters) therefore refer to simulated or implied viewing distances, rather than physically reconstructed 3D distances. For face stimuli, this mapping was anchored to biologically meaningful dimensions, including the average adult interpupillary distance (∼63 mm) and typical face height (∼20 cm). For non-face objects, we selected familiar items (e.g., balls) with canonical real-world sizes (∼20 cm in diameter) comparable to head size. Because both faces and these objects are associated with stable and well-learned size priors, changes in visual angle can be reliably interpreted by observers as changes in viewing distance. To ensure strict experimental control, all stimulus categories (faces and objects) were scaled to follow identical visual angle trajectories over time, thereby equating the physical dynamics of approach across conditions. This design ensures that any differences in neural responses cannot be attributed to differences in low-level expansion dynamics.

Because our primary interest lies in the neural dynamics associated with face approach designed to simulate PS intrusion, it is necessary to compare approaching stimuli against a condition that preserves stimulus identity while removing motion. Therefore, the static condition was included as a critical control to isolate neural activity specifically related to approach dynamics in the PS context, rather than responses driven by stimulus category (e.g., faces) or visual input alone. To achieve this, static stimuli in the EEG experiment were presented at a visual angle of 13.7° (0.98 m), representing the midpoint of the approach trajectory. This choice serves two purposes. First, it ensures that static and dynamic conditions are matched in terms of overall visual input, thereby minimizing confounds related to stimulus size. Second, selecting the midpoint avoids biases associated with very small (far) or very large (near) stimulus sizes, which could differentially influence perceptual or attentional processing. All stimuli were displayed on a 23-inch Dell P2317H monitor (1920 × 1080 resolution, 60 Hz refresh rate) at a viewing distance of approximately 60 cm. Stimulus presentation was controlled using Presentation® software (Version 23.1, Neurobehavioral Systems).

Note that faces are inherently more visually complex and socially salient than non-face objects, and that these properties can influence neural responses. To dissociate their contribution from neural responses specifically associated with approaching stimuli and potential PS intrusion, we employed a 2 (Motion: Approaching, Static) × 3 (Category: Face, Object, Sphere) factorial design. This design allows us to separate (i) category-dependent responses from (ii) modulation induced by approach dynamics. First, within-category contrasts (e.g., Approaching Face vs. Static Face) control for stimulus complexity and identity, isolating the effect of approach for a given category. Thus, any additional modulation observed for approaching faces cannot be attributed solely to their intrinsic complexity or social salience. Second, cross-category comparisons allow us to assess whether motion effects are uniform or category-dependent. Non-social objects and spheres provide controls for structured visual input and low-level expansion, respectively.

#### Behavioral experiment (DD measurement)

To assess participants’ PS preferences, we adapted the computerized Stop-Distance paradigm,^5^ as shown in Figure 1A. Note that the behavioral measure used in this study reflects discomfort distance, which serves as an operational proxy for PS specifically in the context of social stimuli (i.e., faces). For non-social objects, this measure reflects perceived discomfort or tolerance to approach, rather than PS per se. In each trial, participants viewed a video of an approaching stimulus on a computer screen and were instructed to press the spacebar when the stimulus reached a distance at which they felt uncomfortable. Each trial began with a 1000-ms fixation cross, followed by a 5000-ms video of the approaching stimulus presented against a 3D background. In the behavioral task, this 5-second gradual approach was necessary to obtain reliable estimates of discomfort distance using a stop-distance paradigm. The extended trajectory allows participants to continuously monitor the stimulus and respond deliberately, thereby reducing variability associated with reaction-time constraints. Inter-trial intervals (ITIs) were randomized between 1500 and 2000 ms. The stimulus set included videos of different individuals’ faces, as well as objects and spheres serving as controls. The experiment comprised 96 trials (4 exemplars × 3 categories × 8 repetitions). Before the main experiment, participants completed a brief training phase consisting of four practice trials using cartoon images to ensure understanding of the discomfort distance concept. Participants were seated comfortably in a sound-attenuated room for the duration of the experiment, which lasted approximately 15 minutes.

DD was determined by recording the frame number (FN) of the video at which participants pressed a key to indicate discomfort with the approaching stimulus. DD was calculated using the following equation (Equation 1):

(Equation 1) $$DD=550-\left(FN-1\right)\times \frac{550-50}{125-1}$$

where 550 cm and 50 cm represent the maximum and minimum possible distances, respectively, and 125 is the total number of video frames.

For each participant, DD was calculated for each exemplar within every stimulus category and then averaged across the four exemplars to yield a mean DD value for each category.

#### EEG experiment

The EEG experiment employed an event-related potential (ERP) design (Figure 1B). Each trial began with a 1000-ms fixation cross, followed by a 1000-ms presentation of either an approaching or static stimulus against a 3D background. The 1-second approach period in the EEG task was designed to capture neural responses within a temporally constrained window centered on boundary crossing. Importantly, the EEG stimuli were initialized so that the trajectory traversed the typical discomfort boundary identified in the behavioral task. Accordingly, the EEG paradigm can be viewed as a temporally compressed sampling of face approach designed to simulate PS intrusion, rather than as a qualitatively different stimulus from that used in the behavioral experiment. ITIs were randomized between 1500 and 2000 ms. The experiment consisted of four runs, alternating between two runs of approaching stimuli and two runs of static stimuli. Each run comprised six blocks, with each block containing 12 trials (4 exemplars × 3 categories) presented in randomized order. The order of runs was counterbalanced across participants.

While online responses provide explicit behavioral markers, they also introduce substantial motor-related activity, particularly in frontal regions implicated in PS processing. To minimize such confounds and isolate stimulus-driven neural dynamics, the EEG experiment employed a passive viewing paradigm, consistent with prior neuroimaging studies.^18^^,^^24^^,^^53^ This design was chosen because PS processing is thought to involve automatic and affectively salient responses to PS-relevant approaching stimuli,^5^^,^^54^ allowing neural responses to be examined without requiring explicit online distance judgments. Consistent with this rationale, previous PS neuroimaging studies have shown that passive-viewing or non-distance-judgment paradigms can elicit robust neural responses and show meaningful associations with offline behavioral measures.^18^^,^^55^

Participants were instructed to maintain fixation on the central cross and minimize blinking during stimulus presentation. To control for the effects of hand movement, participants passively viewed the stimuli and were not required to respond during the main trials. To ensure sustained attention, 24 catch trials (8 per category) were randomly interleaved among the main trials, with one catch trial per block.^56^ During these trials, red squares appeared at random time points within the videos, and participants were instructed to press the spacebar upon detection. A total of 24 catch trials, which were excluded from further analysis, were evenly distributed across categories (8 catch trials per category) and blocks (one catch trial per block).

Participants whose response rates on catch trials deviated by more than 1.5 standard deviations from the mean were excluded from analysis (specifically, participants S001, S004, S012, and S014). The total duration of the EEG experiment was approximately 45 minutes. The EEG sessions were conducted in the same laboratory environment as the behavioral experiment, using identical stimulus presentation equipment and settings. To prevent adaptation effects, the behavioral and EEG experiments were separated by at least three days.

#### Data acquisition and preprocessing

EEG data were recorded from 64 Ag/AgCl electrodes (Neuroscan) positioned according to the international 10-20 system. Signals were sampled at 1000 Hz using a SynAmps2 amplifier (Neuroscan), and electrode impedances were maintained below 5 kΩ. Eye movements were monitored with vertical (VEOG) and horizontal (HEOG) electrooculograms. AFz served as the ground electrode. During acquisition, data were referenced online to the average of the CPz and Cz electrodes.

EEG data were preprocessed offline using EEGLAB.^57^ The preprocessed data were further analyzed using custom routines implemented in MATLAB (MathWorks, MA, USA). Signals were re-referenced to the linked mastoids (M1 and M2) and band-pass filtered at 0.1-30 Hz for ERP analysis as well as decoding analysis, and at 1-125 Hz for time-frequency analysis, with additional notch filtering at 50 Hz and its harmonics. Note that for Granger causality (GC) analysis, to avoid potential degradation of causal inference caused by pre-filtering stationary data,^50^^,^^58^ we reprocessed the EEG by applying only a notch filter at 50 Hz to remove line noise, followed by downsampling to 500 Hz and performing global referencing. Data were segmented into epochs from -500 to 2000 ms for ERP analysis and from -700 to 2000 ms for time-frequency analysis. A −120 to −20 ms baseline correction was applied, and any bad channels were interpolated. Ocular artifacts were removed using independent component analysis (ICA). One participant (S024) was excluded due to excessive eye movement artifacts.

#### ERP analysis

For each participant, condition, and channel, outlier trials were first identified and excluded if their values exceeded three standard deviations from the cluster centroid, as determined by k-means clustering.^59^ This procedure resulted in the removal of less than 10% of trials.

ERP waveforms were obtained by averaging across participants within each region of interest (ROI), and then peak amplitudes were measured. Based on previous studies,^21^^,^^22^^,^^29^^,^^31^^,^^60^^,^^61^^,^^62^ ERP analyses focused on the P1, N170, and VPP components within predefined ROIs. The P1 was measured at bilateral occipital ROIs (left: O1; right: O2), the N170 at bilateral temporo-occipital ROIs (left: P5, P7, PO5, PO7; right: P6, P8, PO6, PO8), and the VPP at the centro-frontal ROI (FC1-FC4, F1, F2, Fz, C1, C2, Cz). These ROIs were further validated by inspecting topographic maps to confirm that the selected electrodes corresponded to sites of maximal activity. The following time windows were used for component analysis and topographic mapping: 100-140 ms for P1, 155-195 ms for N170 and VPP. These time windows were defined based on the grand-average peak across all categories, conditions, and participants, with a ±20 ms window around the peak.

#### Decoding analysis

Previous fMRI studies have shown that PS processing primarily involves the PMv and the DIPS.^18^ Guided by this literature, we defined sensor-level frontal and parietal electrode clusters, hereafter referred to as ROIs, for hypothesis-driven decoding, time-frequency, and GC analyses. Based on established electrode localization,^63^ the frontal ROI comprised FC1, FC2, FC3, and FC4, while the parietal ROI comprised P1, P2, Pz, and POz.

To assess how neural activity within frontal and parietal ROIs represents different motion directions and stimulus categories, we performed decoding analyses. Specifically, decoding of motion (approaching vs. static) and stimulus category (face, object, sphere) within the predefined frontal and parietal ROIs was conducted using the Neural Decoding Toolbox (version 1.0.4, http://www.readout.info),^48^ libsvm (version 3.25),^49^ and custom MATLAB scripts. For each participant, trials from each of six conditions (2 motion types × 3 categories) were randomly divided into eight splits, with EEG data averaged within each split. A leave-one-out cross-validation procedure was applied using a linear Support Vector Machine (SVM) classifier at each time point. Data were z-score normalized before classification.

Each k-fold partition introduces stochastic variability in training/testing splits, which can affect decoding accuracy in any single run. Repeating the procedure multiple times and averaging the results is a standard practice^64^^,^^65^^,^^66^ to obtain a stable, unbiased estimate of the true decoding performance. We systematically examined the convergence of decoding accuracy as a function of repetition number and found that accuracy stabilized after approximately 15–20 repetitions (Figure S6). Based on this result, we selected 20 repetitions in the main text as a practical and sufficient choice to ensure robustness without incurring unnecessary computational cost. Decoding accuracy was then smoothed using a 50-ms Gaussian kernel.^66^

Note that the use of a restricted feature set (4 channels) in the decoding analysis was hypothesis-driven. As shown in Figure S7, decoding accuracy increased from 2 to 4 channels but plateaued thereafter, with no substantial gains when additional channels were included. Importantly, the temporal characteristics of decoding, including onset latency and peak accuracy, remained highly consistent across feature sets. These results indicate that the 4-channel configuration captures most of the task-relevant neural information while preserving stable temporal dynamics.

#### Time-frequency analysis

To investigate frequency-specific neural dynamics during stimulus processing, we performed time-frequency decomposition on channels within predefined frontal and parietal ROIs. The continuous wavelet transform (CWT) was applied using a Morlet wavelet with a center frequency of 6 Hz and a spread of 0.3, spanning 1-60 Hz in 1 Hz steps. To minimize edge effects inherent to wavelet analysis,^67^^,^^68^ we restricted analyses to post-stimulus periods. After decomposition, outlier trials were identified and excluded for each participant using k-means clustering, applied separately for each condition and each channel, consistent with the ERP procedures. The resulting time-frequency data were then averaged across trials within each condition for each participant. Power values were then z-score normalized relative to a pre-stimulus baseline period from -500 to -100 ms.

#### GC analysis

GC analysis was performed using the Multivariate Granger Causality (MVGC) toolbox^50^ to examine frequency-specific directional interactions between the frontal and parietal ROIs. Time series were extracted from the preprocessed EEG data and segmented into two time windows for each ROI: 1-500 ms, corresponding to the phase in which the calibrated stimuli approached the behaviorally estimated discomfort boundary, and 501-1000 ms, corresponding to the phase after the stimuli had traversed this boundary, thereby simulating intrusion into PS. Prior to GC calculation, each segment was detrended and normalized using ensemble normalization. Stationarity was assessed with the Kwiatkowski-Phillips-Schmidt-Shin (KPSS) test, and non-stationary segments were excluded from further analysis. The optimal model order for each segment and participant was determined via the Akaike Information Criterion (AIC), with a maximum order of 20. Spectral GC curves were then averaged across participants. We conducted permutation statistics by randomly shuffling the time series. If the spectral GC estimates exceeded the 99.9% confidence interval determined by the permutation test (using 1000 randomizations), we considered the estimated causality to be significant.^50^^,^^69^

### Quantification and statistical analysis

All statistical analyses were performed using GraphPad Prism 8 (GraphPad Software, CA, USA) and MATLAB R2020a (MathWorks, MA, USA). Behavioral data were analyzed using the Friedman test, with Category (face, object, sphere) as a within-subject factor, due to violations of normality assumptions. When significant effects were found, post hoc comparisons were performed using Dunn’s test with corrections for multiple comparisons. ERP amplitudes were analyzed using two-way repeated-measures ANOVAs, incorporating Motion (approaching, static) and Category as within-subject factors. The Greenhouse-Geisser correction was applied when sphericity was violated, and Bonferroni-corrected post hoc tests were used to further examine significant main effects and interactions.

Decoding accuracy was assessed against chance levels (50% for motion and 33.3% for category decoding) using a nonparametric cluster-based permutation test with 50,000 permutations. Clusters of above-chance accuracy were identified based on contiguous significant time points (*p* < 0.01, one-tailed), and the cluster-level statistic was compared to the null distribution generated via cluster-based non-parametric permutation testing (50,000 permutations). Clusters were deemed significant if they exceeded the corrected threshold (*p* < 0.01, two-tailed). In this framework, statistical significance depends not only on exceeding the threshold at individual time points, but also on the temporal extent and cumulative strength of contiguous suprathreshold time points (i.e., cluster mass) required for a cluster to survive correction against the permutation-based null distribution.

For time-frequency and GC analyses, we adopted the same cluster-based permutation framework used in the decoding analysis. Specifically, for time-frequency analyses, two-sided paired t-tests were conducted across participants to compare power changes between approaching and static conditions within the 0–1000 ms post-stimulus window for five frequency bands (delta band: 1-4 Hz, theta band: 4-8 Hz, alpha band: 8-13 Hz, beta band: 13-30 Hz, low-gamma band: 32-40 Hz). For GC analysis, two-sided paired t-tests were conducted across participants to compare GC values between approaching and static conditions within two predefined time windows (1–500 ms and 501–1000 ms). For both analyses, data points exceeding an initial uncorrected threshold (*p* < 0.05, two-tailed) were grouped into clusters based on temporal (time-frequency) or spectral (GC) adjacency. Cluster-level statistics were then evaluated against a null distribution generated via 50,000 permutations, and clusters were considered significant if they exceeded the corrected threshold (*p* < 0.05, two-tailed).

Finally, Spearman’s rank correlation (two-tailed) was used to examine associations between PS size and EEG measures.

Unless otherwise specified, n refers to the number of biologically independent participants included in each analysis, with a total sample size of *n* = 28. Statistical tests, exact *p* values, and effect sizes are reported in the results section. Behavioral data are reported as mean ± SD in the results section. Error bars or shaded regions shown in the figures represent either the standard error of the mean (SEM) or 95% confidence intervals (CIs), as specified in the corresponding figure legends. Statistical significance was defined as *p* < 0.05 unless otherwise specified.
