# Supplementary material for: Temporal dynamics of frontoparietal processing during personal space intrusion
Source: iScience. 2026 Jul 16;29(8):116829. doi: 10.1016/j.isci.2026.116829 (PMC13383958; doi:10.1016/j.isci.2026.116829)
Supplement: Document S1. Figures S1–S7 [file mmc1.pdf]

iScience, Volume *29*

Supplemental information

Temporal dynamics of frontoparietal processing during personal space intrusion

Sijia Xiang (项思佳), Xinbo Zou (邹心博), Chuqing Luo (罗楚晴), and Ning Liu (刘宁)

Supplemental Figures

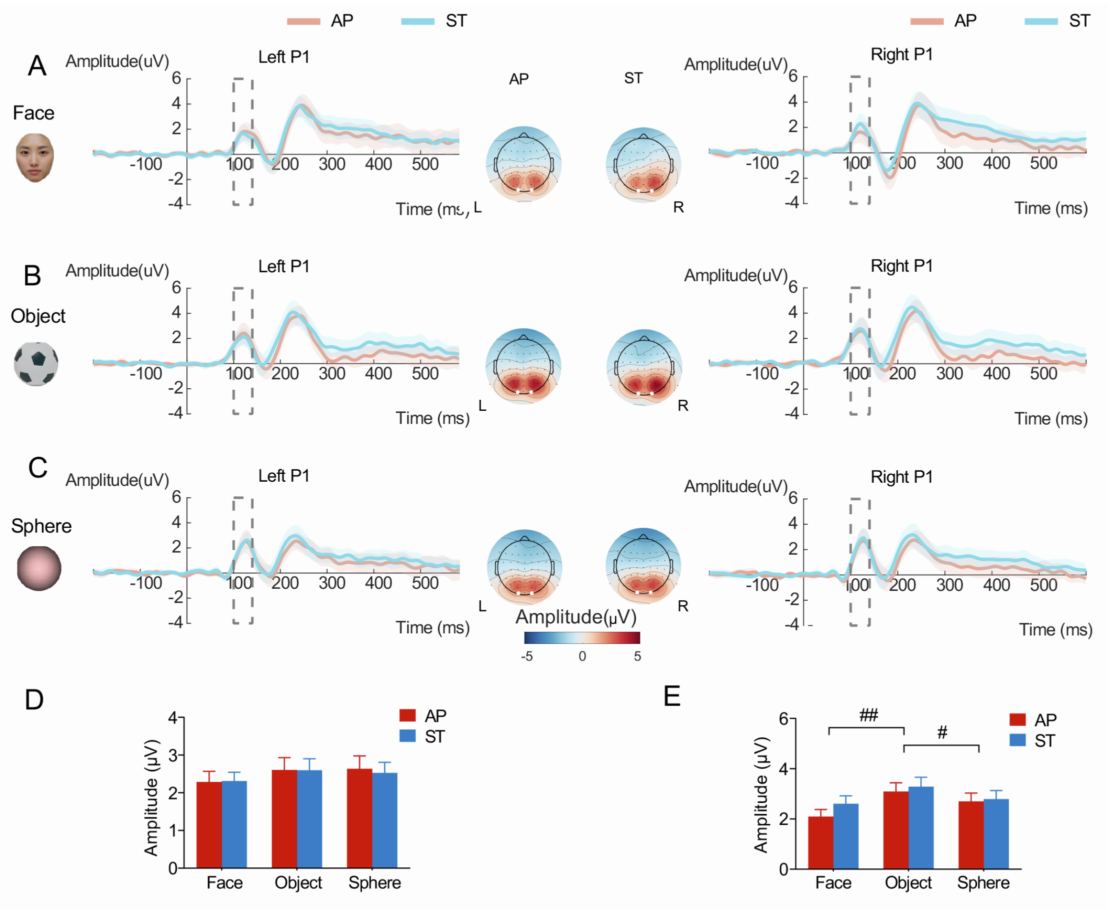

Figure S1. ERP results for the P1, related to Figure 2.

(A-C) Grand-average ERP waveforms for the left and right P1 in response to faces (A), objects (B), and spheres (C). Shaded areas represent 95% CIs. Topographical maps show scalp distributions averaged over the 120–160 ms time window (dashed box in waveforms), with the locations of the electrodes analyzed indicated by white dots. (D, E) Mean amplitudes of the left (D) and right (E) P1 for each condition. Error bars indicate SEM. # indicates follow-up post hoc tests for the main effect of Category. ## $p < 0.01$; $n = 28$ participants, Bonferroni-corrected for multiple comparisons. AP: approaching; ST: static; L: left; R: right.

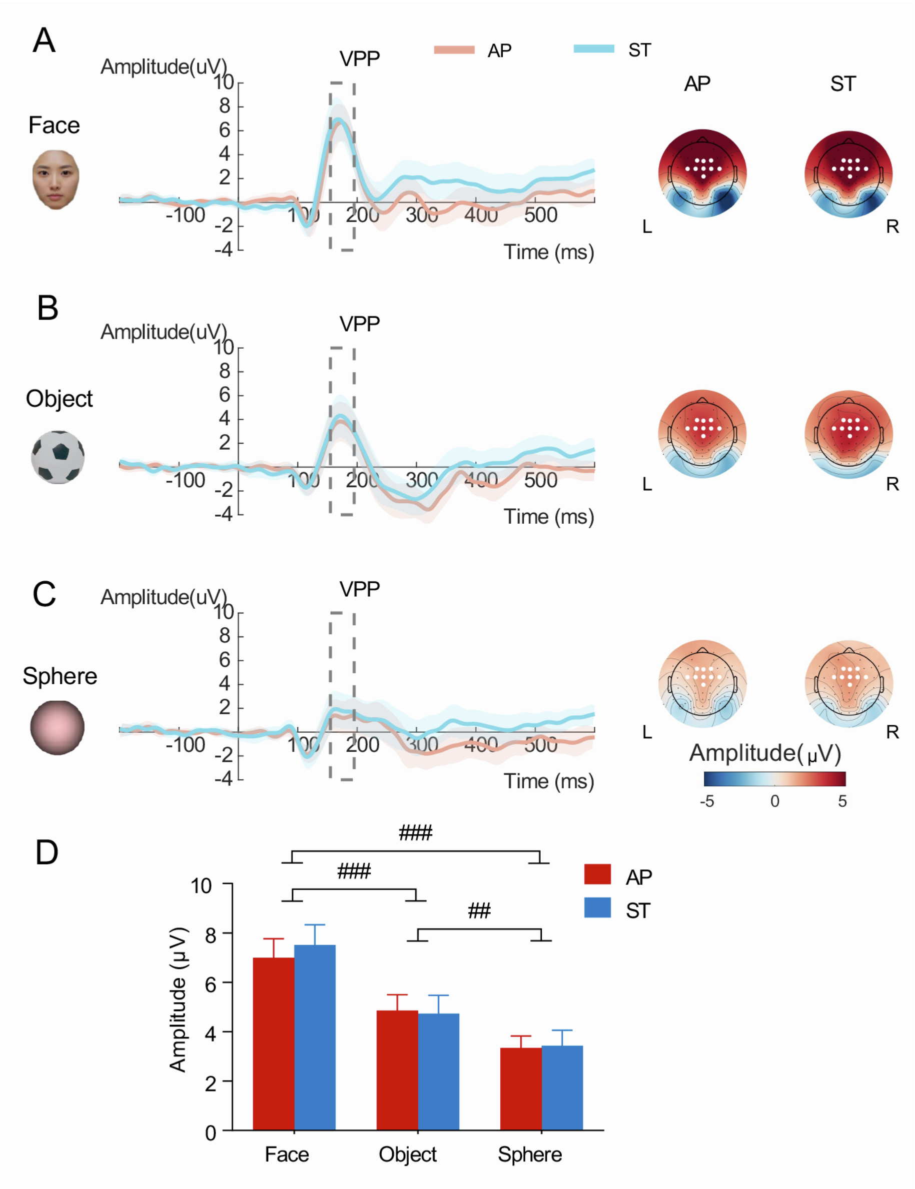

Figure S2. ERP results for the VPP, related to Figure 2.

(A-C) Grand-average ERP waveforms for the VPP in response to faces (A), objects (B), and spheres (C). Shaded areas represent 95% CIs. Topographical maps show scalp distributions averaged over the 175–215 ms time window (dashed box in waveforms), with the locations of the electrodes analyzed indicated by white dots. (D) Mean amplitudes of the VPP for each condition. Error bars indicate SEM. # indicates follow-up post hoc tests for the main effect of Category. ### $p < 0.001$; ## $p < 0.01$; $n = 28$ participants, Bonferroni-corrected for multiple comparisons. AP: approaching; ST: static; L: left, R: right.

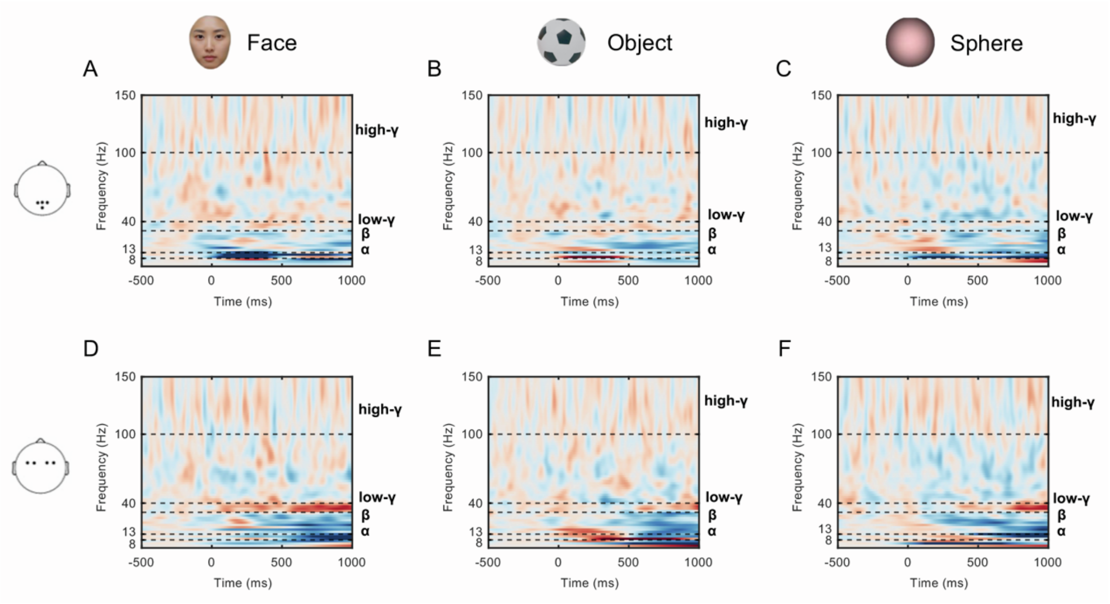

Figure S3. Broadband time-frequency analysis in parietal and frontal ROIs, related to Figure 4.

(A-F) Time-frequency spectrograms contrasting approaching vs. static stimuli in parietal and frontal ROIs for faces (A, D), objects (B, E), and spheres (C, F). Dashed lines delineate the predefined frequency bands. Low-gamma: 32–40 Hz; high-gamma: >100 Hz. n = 28 participants.

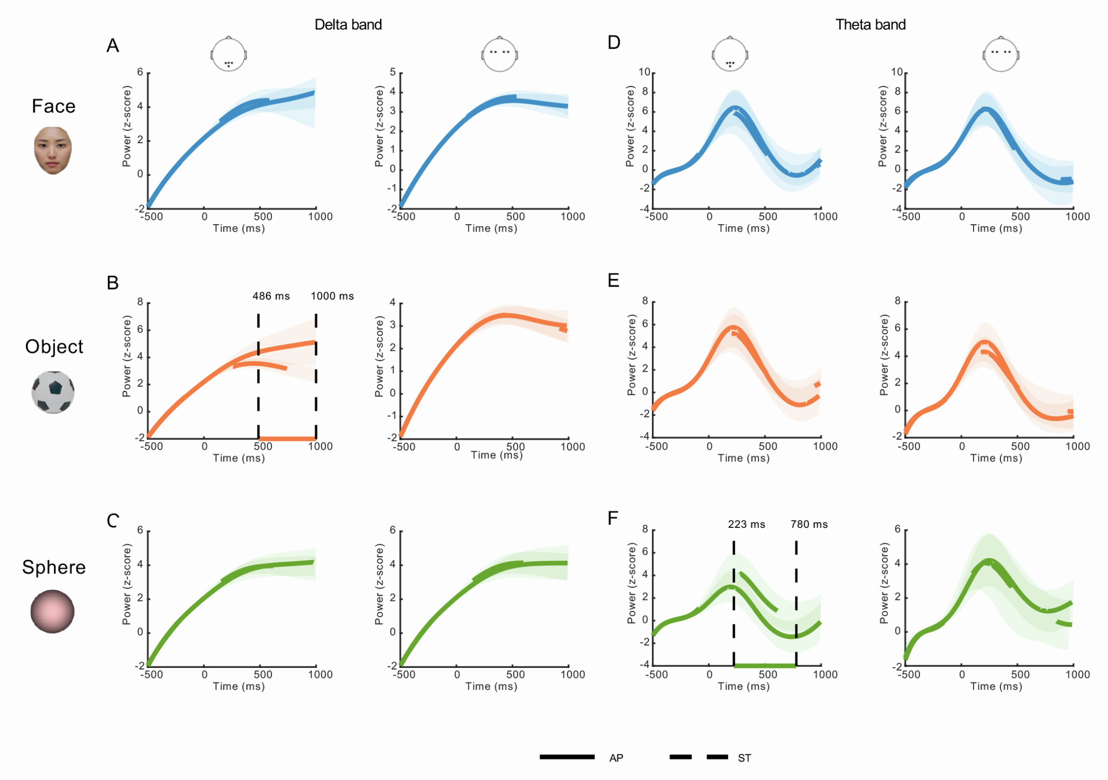

Figure S4. Time-frequency analyses of delta and theta power in parietal and frontal ROIs, related to Figure 4.

(A–C) Delta power averaged over parietal (left) and frontal (right) ROIs as a function of time for faces (A), objects (B), and spheres (C). (D–F) Theta power averaged over parietal (left) and frontal (right) ROIs for faces (D), objects (E), and spheres (F). Solid lines represent the approaching condition; dashed lines represent the static condition. Shaded areas represent 95% CIs. Colored lines overlaid on the x-axis highlight time points with significant differences between approaching and static conditions ($n = 28$ participants; two-sided paired t-tests, cluster-based permutation correction, $p < 0.05$, 50,000 permutations).

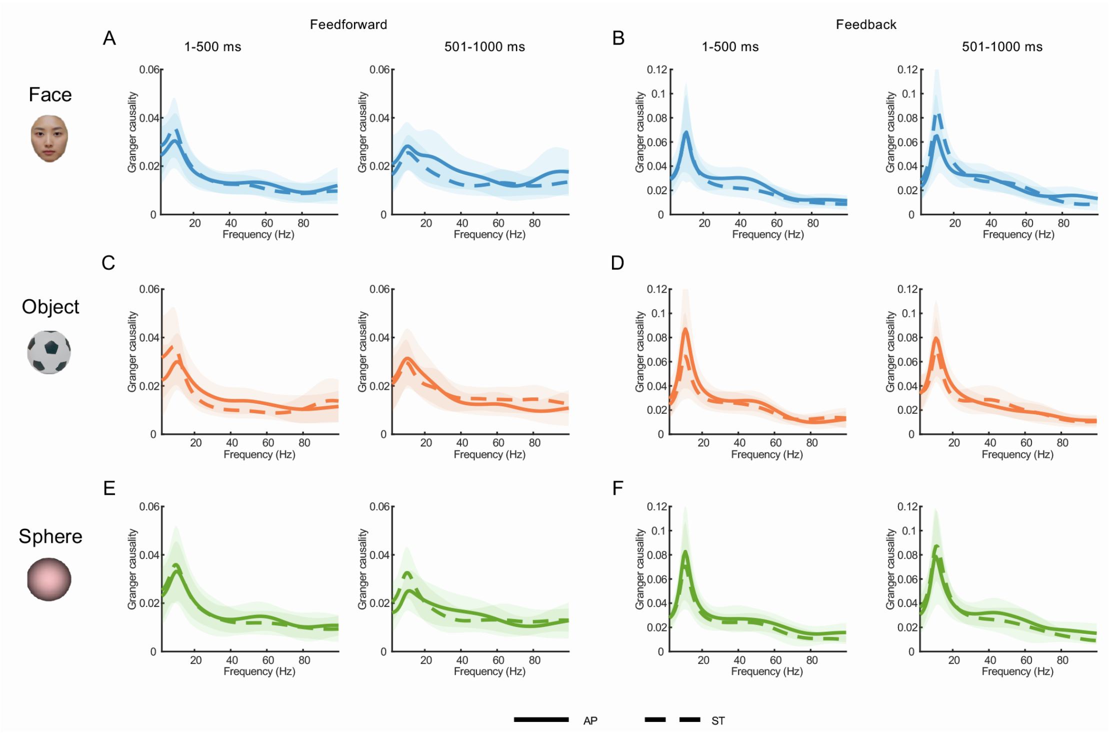

Figure S5. Time-reversed GC analysis between parietal and frontal ROIs, related to Figure 5.

(A–B) Time-reversed spectral GC for feedforward (A) and feedback (B) information in response to faces. (C–D) Time-reversed spectral GC for feedforward (C) and feedback (D) information in response to objects. (E–F) Time-reversed spectral GC for feedforward (E) and feedback (F) information in response to spheres. Solid (approaching) and dashed (static) lines show mean spectral GC; shading indicates 95% CIs. n = 28 participants.

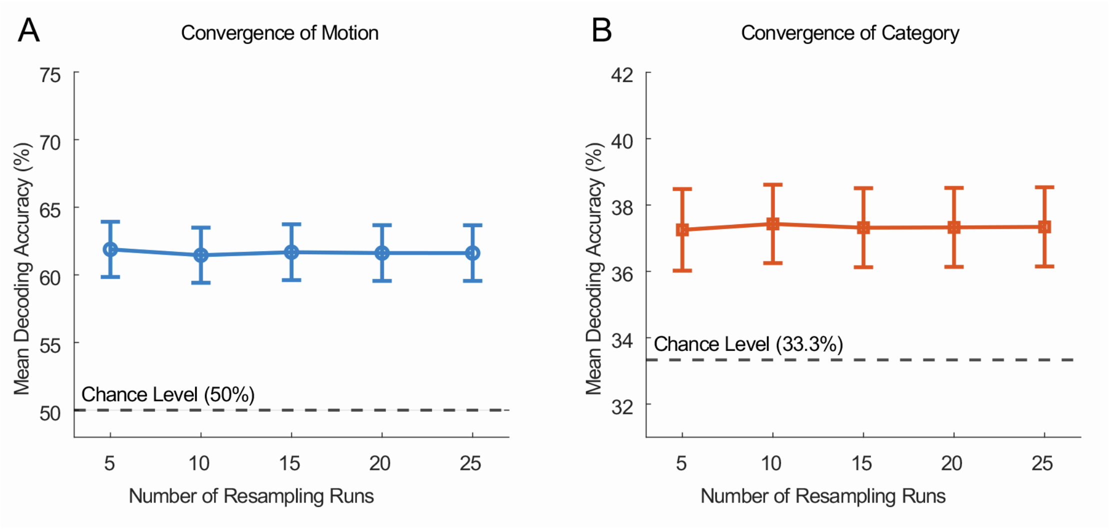

Figure S6. Convergence and stability of decoding performance across multiple cross-validation resampling runs, related to STAR Methods.

(A) Decoding of motion (theoretical chance level = 50%). (B) Decoding of category (theoretical chance level = 33.3%). Mean accuracy was computed by averaging decoding performance across the entire stimulus presentation window (1–1000 ms) for all participants. Error bars represent the SEM. n = 28 participants.

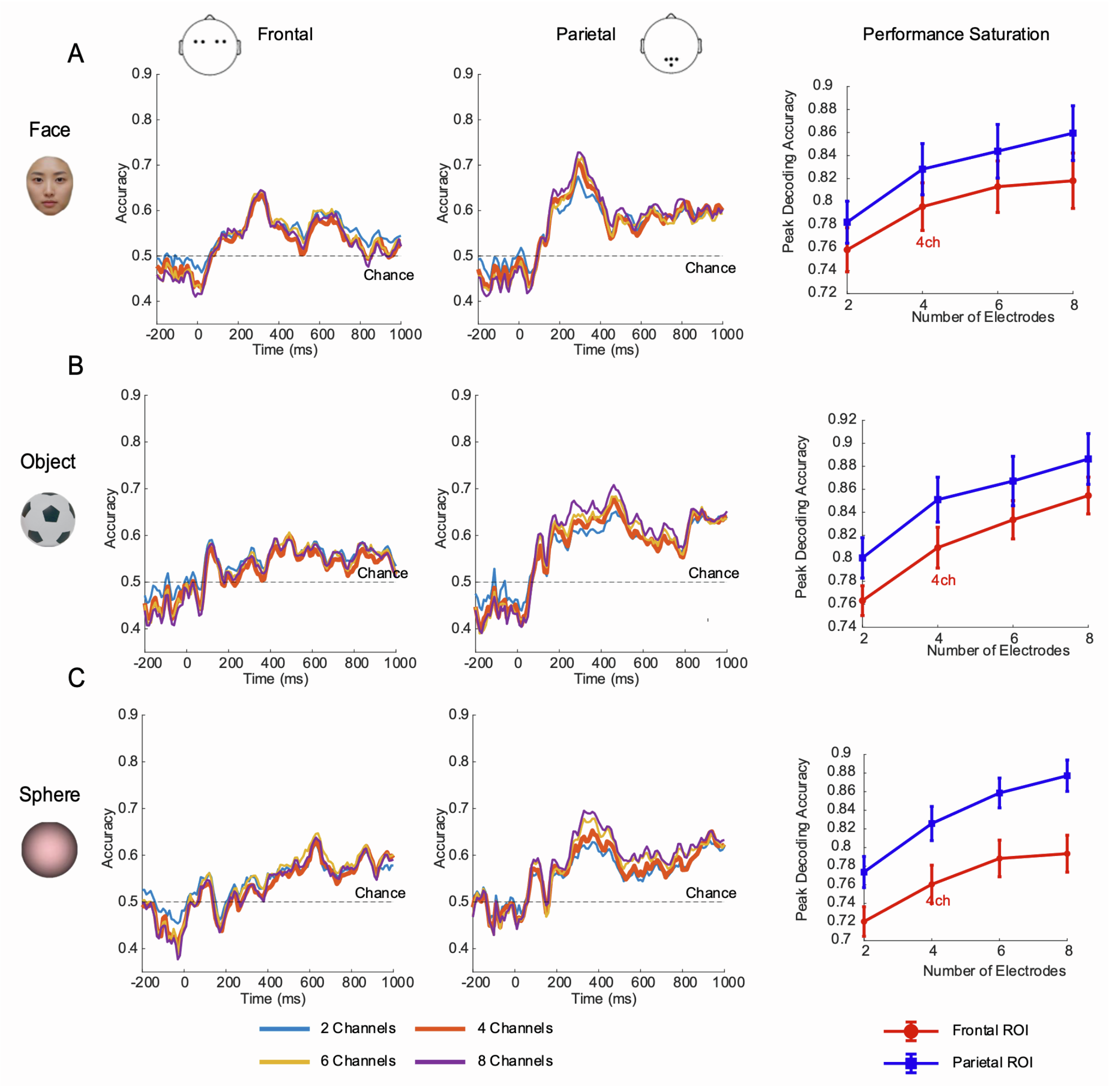

Figure S7. Decoding accuracy across varying feature set sizes, related to STAR Methods.

(A–C) Decoding results for approaching versus static conditions across the three stimulus categories: (A) face, (B) object, and (C) sphere. Left and middle columns: Time-resolved decoding accuracy in the frontal and parietal ROIs, respectively, using 2, 4, 6, and 8 channels as feature vectors. The dashed line indicates chance-level performance. Shaded areas represent 95% CIs. Right column: Peak decoding accuracy (extracted from the 100–600 ms window) as a function of the number of electrodes for the frontal (red) and parietal (blue) ROIs. Error bars represent the standard error of the mean (SEM). n = 28 participants.
